# The ratio of serum free triiodothyronine to free thyroxine in children: a retrospective database survey of healthy short individuals and patients with severe thyroid hypoplasia or central hypothyroidism

**DOI:** 10.1186/s13044-015-0023-5

**Published:** 2015-07-08

**Authors:** Yuji Oto, Koji Muroya, Junko Hanakawa, Yumi Asakura, Masanori Adachi

**Affiliations:** Department of Endocrinology and Metabolism, Kanagawa Children’s Medical Center, Mutsukawa 2-138-4, Minami-ku Yokohama, 232-8555 Japan

**Keywords:** Congenital hypothyroidism, Deiodinase, L-thyroxine therapy, Hypopituitarism, Triiodothyronine, Thyroxine

## Abstract

**Background:**

The ratio of serum free triiodothyronine (FT3) to free thyroxine (FT4) has been shown to be constant in healthy adults. However, this ratio has been found to be decreased in athyreotic adult patients on levothyroxine (L-T4) supplementation. In order to better evaluate thyroid-related pathologies in children as well as to establish a reference range, we investigated the FT3/FT4 ratio in a pediatric population. Furthermore, we evaluated this ratio in children with congenital hypothyroidism as well as those with central hypothyroidism.

**Methods:**

A reference range for the FT3/FT4 ratio was obtained from 129 Japanese children (3–17 y) with idiopathic short stature who were designated as the ‘Control’ group. Patients with congenital hypothyroidism due to athyreosis or severe thyroid hypoplasia (designated as ‘A/Hypoplasia’), as well as patients with central hypothyroidism (‘Central’), were recruited from the institutional database. For each group, the mean FT3/FT4 ratio was obtained.

**Results:**

In the Control group, the FT3/FT4 ratio was 3.03 ± 0.38 10^−2^ pg/ng (mean ± standard deviation) with no age or gender differences. A/Hypoplasia patients showed a significantly decreased mean FT3/FT4 ratio (2.17 ± 0.33, *P* < 0.001) compared to Control patients, with decreased FT3 and elevated FT4 levels. The Central group also showed a significantly decreased FT3/FT4 ratio (2.55 ± 0.45, *P* < 0.001) compared to the Control group, with decreased FT3 and equivalent FT4 levels.

**Conclusions:**

The FT3/FT4 ratio appears to be constant between the ages of 3–17 y. Children on L-T4 due to congenital thyroid a/hypoplasia or central hypothyroidism have a decreased FT3/FT4 ratio compared to short normal children.

## Background

Triiodothyronine (T3) is the most active form of thyroid hormone. While one fifth of net T3 production is performed by the thyroid gland, the remaining 80 % is derived from the extrathyroidal conversion of thyroxine (T4) to T3, which is mediated by either iodothyronine deiodinase 1 or 2 (DIO 1 or DIO 2, respectively). In contrast, T4 is produced exclusively in the thyroid gland [1, 2].

The ratio of serum free T3 (FT3) over free T4 (FT4) (FT3/FT4 ratio) may reflect the degree of extrathyroidal T4 to T3 conversion activity. Whereas FT3/FT4 ratio has been reported to be constant in healthy adult individuals [3], the consistency of this ratio has not yet been clearly determined in the pediatric population.

Abnormally high or low FT3/FT4 ratios are seen in several congenital disorders that may affect peripheral thyroid hormone economy, such as Allan-Herndon-Dudley syndrome (MCT8 deficiency) [4], selenocysteine insertion sequence binding protein 2 deficiency [5], and thyroid hormone resistance [6, 7]. Therefore, investigating the consistency of the FT3/FT4 ratio in the pediatric population, as well as the establishment of a reference range, is of vital importance.

Decreased FT3/FT4 ratios has been observed in postoperative athyreotic adult patients under oral levothyroxine (L-T4) supplementation [3, 8]. This presents the therapeutic problem of setting a target thyroid-stimulating hormone (TSH) range when treating athyreotic populations with L-T4. It also indicates the potential benefit of combination therapy with L-T4 and liothyronine (L-T3). It is therefore probable that these same challenges may apply to children with congenital hypothyroidism, especially those with athyreosis. Additionally, we anticipated that the FT3/FT4 ratio may be unbalanced in children with central hypothyroidism, owing to diminished TSH stimulation on thyroidal T3 production.

The purpose of this study was twofold: First, to investigate any age-dependent variations in the FT3/FT4 ratio in the pediatric population without thyroidal morbidity; and second, to evaluate this ratio in children with congenital hypothyroidism owing to athyreosis or severe thyroid hypoplasia, and in those with central hypothyroidism of any etiology.

## Methods

This study was conducted as a retrospective database survey of the patients who attended the endocrine unit in Kanagawa Children’s Medical Center, the regional children’s hospital near metropolitan Tokyo between April 2004 and March 2010. The Ethics Committee of Kanagawa Children’s Medical Center reviewed and approved the study protocol. Informed consent from the patients and/or their caregivers was not required.

To obtain a reference range for the FT3/FT4 ratio, 129 patients who were diagnosed with idiopathic short stature without any underlying morbidities were selected. Hereafter, this category is referred to as the ‘Control’. They were divided into 5 groups according to their chronological ages: 3–5 y, 6–8 y, 9–11 y, 12–14 y, and 15–18 y. For each interval, mean and standard deviation (SD) of the FT3/FT4 ratio, expressed as 10^−2^ pg/ng, was obtained.

Next, we searched for patients with congenital hypothyroidism due to athyreosis or severe thyroid hypoplasia (designated as ‘A/Hypoplasia’). Thyroid morphology was evaluated by ultrasound imaging and/or scintigraphy with ^123^I or ^99m^TcO_4_^−^. Among all candidate patients, only those who presented with a TSH value greater than 100 μIU/mL during the neonatal period were selected. For each patient, the FT3/FT4 ratio obtained at various ages throughout childhood was extracted from the database only when the concurrently determined TSH level was adequate (0.30–5.0 μIU/mL). If a patient had measured FT3 and FT4 more than once during a chronological year, the mean FT3/FT4 ratio for that age was used.

Finally, patients with central hypothyroidism, whether congenital or acquired, were enrolled; these were designated as ‘Central’. As for patients with growth hormone (GH) deficiency, only GH-replaced patients were included. In each patient, the FT3/FT4 ratio was selected only when the FT4 level was adequate (0.80–1.80 ng/dL). As with congenital hypothyroidism patients, the mean FT3/FT4 ratio at each age was obtained.

During the study period, serum FT3 and FT4 levels were determined by electrochemiluminescence immunoassay (ECLIA) kits: FT3 by Elecsys® FT3II and FT4 by Elecsys® FT4 (Roche Diagnostics, Tokyo, Japan). Antibodies used for FT3 and FT4 determination were ovine monoclonal and polyclonal antibodies, respectively. Serum TSH levels were determined via the ECLIA kit Elecsys® TSH (Roche Diagnostics).

### Statistical analysis

Statistical analysis was performed using the SPSS software (Version 16.0; SPSS, IL, Chicago, USA). Data were compared using paired *t*-tests with normal distributions. Differences between groups were evaluated by the covariance analysis test. FT3/FT4 ratios lower than −2 SD or higher than +2 SD were regarded as outliers. A *P* value less than 0.05 was considered significant.

## Results

The profiles of the 129 children with idiopathic short stature (the Control group) are shown in Table 1. Table 2 shows the reference ranges for the FT3/FT4 ratio in each age group, together with individual FT3 and FT4 values, obtained from the Control group. The FT3/FT4 ratio was constant across each age interval, although a significant decrease of FT3 was observed in the 12–14 y age group compared to the 6–8 y age group (*P* = 0.037). There was no significant difference between males and females in the Control group (Table 3).

**Table 1:** Profiles of the three categories of patients enrolled in this study

Category^a^	n (male)	Age range at study in years	Median latest L-T4^b^ dose (range)	Total number of FT3 and FT4 measurements^c^	Mean TSH^d^ level (range)
Control	129 (81)	3.1–17.1	Not applicable	129	2.16 ± 1.03 (0.56–4.96)
A/Hypoplasia	22 (11)	3.0–18.4	125 (25–200)	156	1.81 ± 1.19 (0.30–4.96)
Central	27 (13)	3.0–18.9	100 (50–200)	366	Not applicable

^a^See text for the definition of each category

^b^L-T4: L-thyroxine, in μg per day

^c^These numbers represent all the individual FT3 and FT4 measurements taken before averages were calculated. In A/Hypoplasia and Central, the values exceed the number of patients owing to repetitive measurements

^d^Values are μIU/mL; mean ± standard deviation

**Table 2:** FT3 and FT4 levels, as well as their ratios, in patient groups according to age

		3–5 y	6–8 y	9–11 y	12–14 y	15–18 y	Total
Control (*n* = 129)	n	24	19	31	36	19	129
	FT3	4.11 ± 0.56	4.29 ± 0.43^α^	4.14 ± 0.38	3.89 ± 0.50^α^	3.93 ± 0.56	4.06 ± 0.50
	FT4	1.36 ± 0.14	1.38 ± 0.11	1.36 ± 0.14	1.33 ± 0.16	1.31 ± 0.10	1.35 ± 0.14
	FT3/FT4	3.02 ± 0.38	3.12 ± 0.31	3.07 ± 0.38	2.95 ± 0.35	3.01 ± 0.50	3.03 ± 0.38
A/Hypoplasia (*n* = 22)	n^b^	14	20	22	21	24	101
	FT3	4.15 ± 0.51	3.86 ± 0.42	3.91 ± 0.45	3.84 ± 0.60	3.52 ± 0.60	3.82 ± 0.55*
	FT4	1.95 ± 0.20**	1.81 ± 0.31**	1.76 ± 0.26**	1.85 ± 0.59**	1.90 ± 0.69**	1.85 ± 0.47**
	FT3/FT4	2.15 ± 0.25**	2.18 ± 0.30**	2.25 ± 0.39**	2.23 ± 0.34**	2.03 ± 0.30**	2.17 ± 0.33**
Central (*n* = 27)	n^b^	11	31	46	31	26	145
	FT3	3.50 ± 0.48	3.49 ± 0.53**	3.51 ± 0.64**	3.63 ± 0.64	3.46 ± 0.54	3.52 ± 0.58**
	FT4	1.24 ± 0.23	1.31 ± 0.17	1.43 ± 0.29	1.45 ± 0.30	1.49 ± 0.25	1.41 ± 0.27
	FT3/FT4	2.94 ± 0.58	2.68 ± 0.51*	2.49 ± 0.37**	2.52 ± 0.37*	2.37 ± 0.40**	2.55 ± 0.45**

Values are pg/mL (FT3) and ng/dL (FT4); mean ± standard deviation

^α^ *P* < 0.05, *P < 0.05; **P < 0.01, compared to control, respectively

^b^In A/Hypoplasia and Central, patients underwent repetitive measurements of FT3 and FT4. All the values are averaged according to the chronological age of the patients. The same patients may have average independent measurement values in more than one age category if they were evaluated over multiple years

**Table 3:** Gender difference of FT3/FT4 ratio in Control

		3–5 y	6–8 y	9–11 y	12–14 y	15–18 y	Total
Male	n	10	11	19	29	12	81
	FT3/FT4	3.03 ± 0.27	3.23 ± 0.32	3.06 ± 0.30	3.02 ± 0.34	3.09 ± 0.37	3.06 ± 0.32
Female	n	14	8	12	7	7	48
	FT3/FT4	3.02 ± 0.0.46	2.97 ± 0.25	3.08 ± 0.50	2.83 ± 0.16	3.11 ± 0.23	3.02 ± 0.40

Values are mean ± standard deviation

There were 22 patients with congenital hypothyroidism due to A/Hypoplasia with neonatal TSH levels greater than 100 μIU/mL (Table 1). A total of 101 sets of FT3 and FT4 values obtained with adequate TSH levels (0.30–5.0 μIU/mL) from these patients were utilized for FT3/FT4 ratio calculation.

**Fig. 1:**
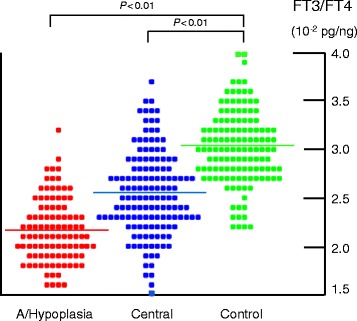
Serum free triiodothyronine (FT3) to free thyroxine (FT4) ratio distribution in the Control, A/Hypoplasia, and Central groups. In the A/Hypoplasia and Central groups, the number of dots exceeds the patients’ number because all the individual FT3/FT4 measurements with averaging per chronological age groups are included. Note the significantly decreased FT3/FT4 ratio in both the A/Hypoplasia and Central groups compared to the Control group

Results of FT3/FT4 ratio analysis in A/Hypoplasia patients, with their respective FT3 and FT4 values, are presented in Table 2 and Fig. 1. For all age intervals, the FT3/FT4 ratio was significantly lower than that of the respective Control, whereas the FT4 levels in A/Hypoplasia were significantly higher than that of the Control. Although there was no difference in FT3 levels between A/Hypoplasia and Control when comparing within each age interval, the difference was significant when compared as a whole group (*P* = 0.004, Table 2).

There were 27 patients in the Central group who were GH sufficient or replete (Table 1). Of these, 11 patients had congenital hypopituitarism (including 5 with septo-optic dysplasia) and 16 had acquired hypopituitarism (11 with craniopharyngioma, 4 with germinoma, and 1 with ROHHADNET syndrome). A total of 145 sets of values for FT3 and FT4 (where FT4 levels were 0.80–1.80 ng/dL) were utilized for FT3/FT4 ratio calculation.

In Table 2 and Fig. 1, the FT3 and FT4 values, as well as the FT3/FT4 ratio, in the Central group are also presented. The total FT3/FT4 ratio was significantly lower than that of the Control group, as was the case in all individual age groups except for the youngest. The FT4 value in the Central group was not different than that of the Control group, whereas the total FT3 value of the Central group was significantly lower than that of the Control group (*P* = 0.02).

Table 4 depicts a schematic summary of the FT3/FT4 ratio, as well as the levels of FT3 and FT4, in the A/Hypoplasia and Central groups compared to the Control group.

**Table 4:** Summary of FT3/FT4 ratio and individual levels in congenital hypoplasia and central hypothyroidism

	Congenital thyroid hypoplasia^a^	Central hypothyroidism^a^
FT3/FT4 ratio	↓↓	↓
FT3	↓	↓↓
FT4	↑↑	→

^a^Compared to the control group

## Discussion

We observed a marked consistency in the FT3/FT4 ratio (mean, 3.03 ± 0.38) among healthy short Japanese children between 3–17 years of age, with no differences owing to gender. This finding is in line with a previous study conducted in an adult population by Gullo et al. [3], which reported a consistent FT3/FT4 ratio with a median molar ratio of 0.32 (corresponding to 2.71 10^−2^ pg/ng), in 3,875 euthyroid subjects (with minor age and gender variability). The difference in the absolute value of the ratio between Gullo et al.’s data [3] and ours may be multifactorial, owing to both the methodological differences in hormone detection (particularly the types of the antibodies used) and ethnic differences that influence iodine sufficiency.

Our report is the first to specifically investigate the FT3/FT4 ratio in the pediatric population. While reference values for FT3 and FT4 in children have been reported [9–13], these reports did not refer to the FT3/FT4 ratio. Additionally, most studies provided only the ranges or centile values of FT3 and FT4, and calculation of the FT3/FT4 ratio was therefore impractical. More research is needed to evaluate the distribution of the FT3/FT4 ratio in both the longitudinal and cross-sectional aspects. The influence of pubertal development on this ratio also ought to be investigated, because FT3 levels in the 12–14 y groups were found to be lower than in the younger age groups.

The consistency of the FT3/FT4 ratio during childhood is useful for the screening of several genetic disorders related to peripheral thyroid hormone metabolism, including Allan-Herndon-Dudley syndrome (MCT8 deficiency) [4], selenocysteine insertion sequence binding protein 2 deficiency [5], thyroid hormone resistance [6, 7], and others.

Furthermore, evaluating the FT3/FT4 ratio in patients on L-T4 supplementation due to various thyroidal disorders may be a method of assessing the sufficiency of the L-T4 therapy. Observing differences of FT3/FT4 ratios in pathological situations was the main feature of this study, which is the first to evaluate this ratio in pediatric patients with congenital hypothyroidism and central hypothyroidism.

The FT3/FT4 ratio in patients with A/Hypoplasia, which is defined by extremely high neonatal TSH levels (>100 μIU/mL), was significantly lower than that in the Control group (Table 2 and Fig. 1). As depicted in Table 4, this decrease is mainly due to the elevation of the denominator (FT4 level), although a slightly decreased numerator (FT3 level) also contributed. This finding is consistent with recent studies conducted in adult populations. For example, Ito et al. found that athyreotic adult patients (*n* = 51) who underwent total thyroidectomy for papillary thyroid carcinoma and who are on L-T4 therapy with normal TSH level (0.3–5 μIU/mL) had increased FT4 and slightly decreased FT3, resulting in a decreased FT3/FT4 ratio [8]. Additionally, Gullo et al. reported that 29.6 % of 1,811 patients who were thyroidectomised because of thyroid cancer and were on L-T4 therapy with TSH levels of 0.4–4.0 μIU/mL, had FT3/FT4 ratios below the lower limit of euthyroid controls [3].

Elevated FT4 levels in our A/Hypoplasia group and athyreotic adult patients is expected because, in athyreotic patients, thyroid hormone is solely derived from exogenous L-T4 whereas thyroidal T3 production is almost absent. Thus, normalization of TSH is achieved at the expense of higher FT4 levels. This is the inverse situation of the increased FT3/FT4 ratio in untreated or refractory Graves’ disease, where thyroidal T3 production is predominant [14].

Ito et al. explained the pathophysiology of decreased FT3 levels in their athyreotic adult patients as follows [8]: First, TSH secreting cells have T3 in their cytoplasm transported directly from the circulation. Second, these cells also have T3, which is generated from T4 by intracellular deiodinase. Third, referring to a classical study [15], it was shown that the proportion of the latter T3 is increased in hypothyroid patients on L-T4. Thus, when target TSH levels are set to approximate the physiologically normal range, circulating FT3 will be maintained at lower level. As Gullo et al. pointed out, the reason for this phenomenon is the inadequacy of peripheral T3 production, which cannot compensate for the absent thyroidal T3 production [3]. This same scenario likely applies to our A/Hypoplasia patients.

The Central group was also found to have a significantly decreased FT3/FT4 ratio compared to the Control group. This decrease is mainly due to grossly decreased FT3 levels with maintained FT4 levels, resulting in a less prominent decrease of the ratio than that found in the A/Hypoplasia group (Table 2 and Fig. 1). There does not appear to be a precedent study investigating the FT3/FT4 ratio in subjects with central hypothyroidism in either adult or pediatric populations.

The explanation for decreased FT3/FT4 values in the Central group is straightforward: As TSH level cannot be used as a therapeutic guide in patients with central hypothyroidism [16], they instead undergo L-T4 therapy to achieve normal FT4 levels. Thus, FT3 levels must be lower because T3 is generated mainly from the peripheral conversion of exogenous L-T4 to T3, and thyroidal T3 production is diminished under attenuated TSH stimulation.

The clinical significance of the decreased FT3/FT4 ratio in the A/Hypoplasia and Central groups is unclear. It has been reported that 5–10 % of hypothyroid patients treated with L-T4 therapy suffered from symptoms indicative of persistent hypothyroidism such as impaired well-being, diminished cognitive functions, anxiety, and depression despite adequate TSH suppression [17–19]. Whether these symptoms are related to abnormal FT3/FT4 ratios is unknown. In clinical trials, L-T4 and L-T3 combination therapy was found to be beneficial in terms of quality-of-life and the severity of depression, and was also found to ameliorate tissue hypothyroidism [20, 21]. Alternatively, Ito et al. suggested that TSH-suppressive doses of L-T4 are required to achieve preoperative native serum FT3 levels in patients who have undergone total thyroidectomy [8]. Further studies are required to determine the significance of decreased FT3/FT4 ratios and their effects of therapeutic interventions [17].

The main drawback of this study is that the Control group constituted children with normal short stature. Although it is clearly more ideal to compare our subjects to the general population, we could not evaluate thyroid function in such subjects because of ethical limitations. In the pediatric field, however, other studies have utilized data obtained from normal short children as references [22, 23]. Additionally, all patients in the Control group underwent thorough evaluation for possible underlying diseases, including hypothalamic-thyroid axis. Therefore, we anticipate that the data from the Control group can be utilized as an alternative to currently used references.

## Conclusions

The FT3/FT4 ratio appears to be constant between 3–17 y. Pediatric populations with congenital hypothyroidism due to severe thyroidal hypoplasia and with central hypothyroidism with exogenous L-T4 supplementation were found to have decreased FT3/FT4 ratios compared to short normal children. The clinical significance of this finding should be further investigated.

## Abbreviations

TSH: Thyroid stimulating hormone
T3: Triiodothyronine
FT3: Free triiodothyronine
T4: Thyroxine
FT4: Free thyroxine
L-T3: Liothyronine
L-T4: Levothyroxine
SD: Standard deviation
